# Supplementary material for: Reverse dark current in organic photodetectors and the major role of traps as source of noise
Source: Nat Commun. 2021 Jan 22;12:551. doi: 10.1038/s41467-020-20856-z (PMC7822930; doi:10.1038/s41467-020-20856-z)
Supplement: Supplementary file 1 — Supplementary Information. [file 41467_2020_20856_MOESM1_ESM.pdf]

# Supplementary Information for:

# Reverse Dark Current in Organic Photodetectors and the Major Role of Traps as Source of Noise

Jonas Kublitski[1*], Andreas Hofacker[1*], Bahman K. Boroujeni[2,3], Johannes Benduhn[1], Vasileios C. Nikolis[1,4], Christina Kaiser[5], Donato Spoltore[1], Hans Kleemann[1], Axel Fischer[1], Frank Ellinger[2,3], Koen Vandewal[6*], Karl Leo[1,3]

[1]Dresden Integrated Center for Applied Physics and Photonic Materials (IAPP) and Institute for Applied Physics, Technische Universität Dresden, Nöthnitzer Str. 61, 01187 Dresden, Germany

[2]Chair of Circuit Design and Network Theory (CCN), Technische Universität Dresden, 01069 Dresden, Germany

[3]Center for Advancing Electronics Dresden (cfaed), Technische Universität Dresden, 01062 Dresden, Germany

[4]Heliatek GmbH, Treidlerstrasse 3, 01139 Dresden, Germany

[5]Swansea University, Singleton Park SA2 8PP, Wales, UK

[6]Instituut voor Materiaalonderzoek (IMO), Hasselt University, Wetenschapspark 1, BE-3590, Diepenbeek, Belgium

[*]Corresponding authors: jonas.kublitski@tu-dresden.de, andreas.hofacker@tu-dresden.de, koen.vandewal@uhasselt.be

# Contents


1 Supplementary Note 1. Device Optimization for Dark Current Studies . . . . . 3

1.1 Contact Selectivity and Blocking Layers . . . . . . . . . . . . . . . . . . 4

1.2 Shunt Paths in OPDs . . . . . . . . . . . . . . . . . . . . . . . . . . . . 5

1.3 Device Structuring . . . . . . . . . . . . . . . . . . . . . . . . . . . . . 6

2 Supplementary Note 2. Reconstruction of the Trap Density of States via Impedance Spectroscopy . . . . . . . . . . . . . . . . . . . . . . . . . . . 7

3 Supplementary Note 3. Impedance Spectroscopy in Organic Blends . . . . . . 8

4 Supplementary Note 4. Generation-Recombination Statistics and Drift-Diffusion Modeling . . . . . . . . . . . . . . . . . . . . . . . . . . . . . . . . . . . . 11

5 Supplementary Note 5. Noise Measurements . . . . . . . . . . . . . . . . . . 13

6 Supplementary Tables . . . . . . . . . . . . . . . . . . . . . . . . . . . . . 15

7 Supplementary Figures . . . . . . . . . . . . . . . . . . . . . . . . . . . . 20

**Supplementary References** **30**

# 1 Supplementary Note 1. Device Optimization for Dark Current Studies

In this section, we discuss a series of optimizations that have been performed to disclose the dependence of $J_D$ on $E_{CT}$. As discussed in the main text, many effects can hide this dependence, since different effects increase the leakage current. The device structure used in each section is depicted in Supplementary Table 1. The final device structure of each of the following results corresponds to a glass substrate coated with structured ITO(Thin Film Devices Inc., 90 nm)/p-layer/i-layer/$C_{60}$ (20 nm, buffer layer)/n-layer/Al (100 nm). After optimization, the final structure used within the main text is **OPD 7**, highlighted in green.

**Supplementary Table 1: Structures used to study different aspects of $J_D$ in the following sections** | The devices were produced by thermal evaporation on a glass substrate coated with structured ITO/p-i-n (as described in the table)/$C_{60}$ (20 nm, buffer layer)/Al (100 nm).

| | p-layer | | | | i-layer | | n-layer | | | |
|---|---|---|---|---|---|---|---|---|---|---|
| | HTL | | EBL | | active layer | | ETL | | HBL | |
| | Material[a] | Thickness (nm) | Material | Thickness (nm) | Donor | Concentration (wt%) | Material[b,c] | Thickness (nm)[d] | Material | Thickness (nm)[e] |
| OPD 1 | p-MeO-TPD | 20 | MeO-TPD | 20 | ZnPc | 50 | variable | 8 or 10 | variable | 0 or 10 |
| OPD 2 | | 20 | | 20 | TPDP | 5 | variable | 8 or 10 | variable | 0 or 10 |
| OPD 3 | | 20 | | variable | ZnPc | 50 | n-HATNA-$Cl_6$ | 10 | HATNA-$Cl_6$ | 5 |
| OPD 4 | | 20 | | 20 | ZnPc | 50 | | 10 | | variable |
| OPD 5 | | 20 | | 20 | TPDP | 5 | | 10 | | 5 |
| OPD 6 | | 20 | | 20 | TAPC | 5 | | 10 | | 5 |
| OPD 7 | | 20 | | 20 | Donor | 5 | | 10 | | 5 |

[a] $F_6$-TCNNQ dopant used at 1 wt%.
[b] $W_2(hpp)_4$ dopant used at 7 wt%.
[c] Bphen used undoped.
[d] 8 nm referrers to BPhen, 10 to the other materials.
[e] 0 nm referrers to BPhen, 10 to the other materials

## 1.1 Contact Selectivity and Blocking Layers

When biasing the device in reverse direction, the selectivity of the contacts is very important. Selectivity means how efficiently the injection of the wrong charge carriers (electrons into p-type contact and holes into n-type contact) is blocked. Generally, this property should be controlled by the energy levels of the materials. However, in some cases, different effects can also play a role.

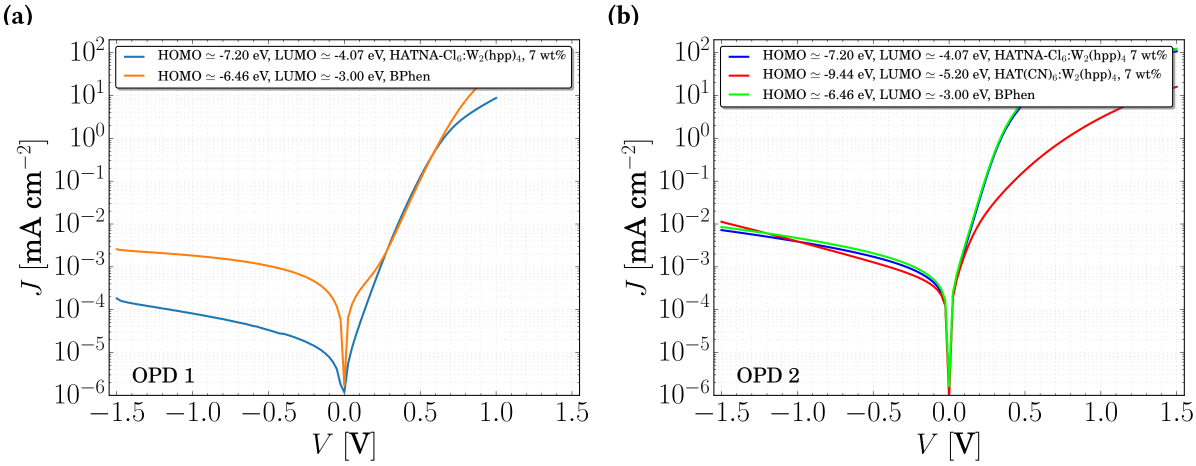


**Supplementary Figure 1: Dark current with different electron transport layers and for different donor** | In **(a)** ZnPc is used as donor blended with $C_{60}$ (50 wt%), in **(b)** TPDP (5 wt%) is the donor, also blended with $C_{60}$. The intrinsic dark current generated in the TPDP:$C_{60}$ bulk heterojunction (BHJ) is higher than the effect of bad selectivity of BPhen, clearly observed in the ZnPc:$C_{60}$ BHJ.

In Supplementary Figure 1a and Supplementary Figure 1b, we show $JV$ characteristics comprising ZnPc and TPDP as donors, respectively. Using p-HATNA-$Cl_6$ in ZnPc:$C_{60}$ bulk heterojunction (BHJ) reduces $J_D$ considerably. This effect is caused by a better selectivity of the p-doped layer, in comparison to BPhen. Interestingly, the same is not true for the TPDP BHJ. This indicates that, in the case of TPDP, another mechanism dominates $J_D$ and the minor effect of selectivity is no longer observed. Moreover, because it has a very low ionization potential (IP), which should express a better selectivity, HAT(CN)$_6$ was also employed as n-doped layer, as further comparison. Once more, no improvements are observed reinforcing the secondary character of the selectivity for this material system.

Comparing IPs of HATNA-$Cl_6$ and BPhen, -7.20 eV[1] and -6.46 eV[2], respectively, one can already conclude that the selectivity should be better for HATNA-$Cl_6$. In addition, BPhen should cause an extraction barrier in forwards bias, when used with $C_{60}$, as its electron affinity (EA) is shallower in energy. However, this is not observed for thin layers ($\leq$ 8 nm). Therefore, it is reasonable to assume that a thin BPhen layer gets doped by the metal[3] or that trap states are formed within its gap, allowing not only electron extraction in forward direction, but also hole injection in reverse bias, explaining the bad selectivity observed in Supplementary Figure 1a.

Similarly to the selectivity property, another commonly used strategy to reduce $J_D$ is the use of blocking layers. For blocking electron (holes) injection in reverse direction, the material

should have as low (high) as possible EA (IP) level. This relies once more in the energy levels of these materials. By itself, the energy levels of n-HATNA-$Cl_6$ and p-MeO-TPD should comply this role. Besides, we have inserted a further layer of neat HATNA-$Cl_6$ and MeO-TPD as hole blocking layer (HBL) and electron blocking layer (EBL), respectively.

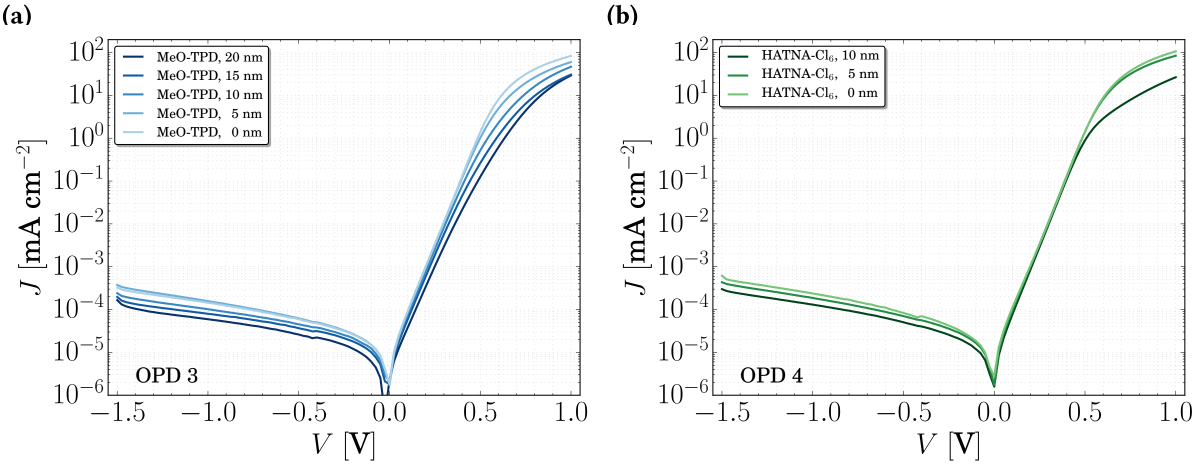


**Supplementary Figure 2: Dark currents of devices with different blocking layers thickness |** **(a)** MeO-TPD is used as EBL and varied from 0 to 20 nm. **(b)** HATNA-$Cl_6$ is employed as HBL using different thicknesses. $J_D$ is reduced for both cases but due to the lower conductivity of these undoped layers, the forward region is also affected.

The results shown in Supplementary Figure 2 indicate that the insertion of a further blocking layer slightly improves the dark current, in both sides of the device. These layers, however, can also affect the behavior in forward bias, as can be seen for thicker layers of neat HATNA-$Cl_6$ and MeO-TPD. Note that the curves indicated as "0 nm" still contain the doped layers, which also presents blocking properties, as discussed above.

## 1.2 Shunt Paths in OPDs

The presence of shunt paths in thin-film devices is well-known in the literature[4,5]. Shunt usually refers to ohmic metallic paths formed between the top and bottom contact, but it may also be understood as an easier path that might be followed by charge carriers than the rectifying diode path[6]. Since the active layer is sandwiched between buffer and/or blocking layers in our device, the latter is assumed to be very unlikely. Moreover, p-i-n devices, using doped layers, should also contribute in this sense, due to the good selectivity of the contacts achieved by doping[7]. Hence, we mainly investigate the presence of metallic paths, which could lead to high dark currents, especially in reverse bias.

In Supplementary Figure 3 we show $JV$ characteristics for four different active layer thicknesses using the same optimized structure and TPDP:$C_{60}$ as BHJ. The thickness of the active layer is varied from 50 nm to 200 nm. If shunts would be present in the device, it is likely that $J_D$ would decrease upon thickness increase. As can be seen, $J_D$ does not show any trend within

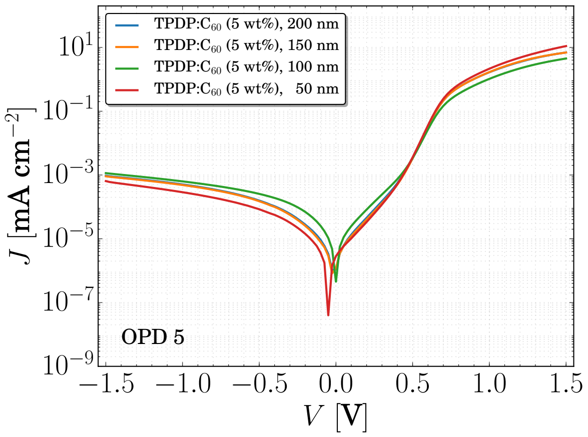


**Supplementary Figure 3: Dark currents versus active layer thicknesses and the role of device structuring** | Four different thicknesses are used, varying from 50 nm to 200 nm. No reduction of $J_D$ is observed upon increasing the thickness. In fact, the device with 50 nm shows the best dark current.

this range and the lowest value is achieved for 50 nm. Supplementary Figure 3 supports the fact that shunts are not responsible for high $J_D$ observed in these devices.

## 1.3 Device Structuring

The last topic investigated within this optimization is the influence of the lateral leakage current, as discussed by Zheng *et al.*[8]. The doped layers, namely n-HATNA-$Cl_6$ and p-MeO-TPD, were structured following the same approach discussed by the authors. The devices discussed in this work are formed by organic materials sandwiched between two crossed-like electrodes: a pre-structured ITO layer as anode and a metal layer cathode. The organic materials are deposited over a larger area than that of the actual device area. Due to the high conductivity of doped layers, lateral leakage current might flow from the surroundings and be collected as dark current. This means that in fact the real device area could be larger than 6.44 $\mathrm{mm}^2$ and undefined.

By structuring the doped layers using shadow masks, Zheng *et al.* were able to reduce the dark current of OLEDs by at least two orders of magnitude. More details about the structuring procedure can be found elsewhere[8]. Our results employing this approach are shown in Supplementary Figure 4. Interestingly, the effect of structuring can be clearly observed for TAPC:$C_{60}$ devices, however, for TPDP:$C_{60}$ this is not the case, where $J_D$ remain unaffected. This result shows that lateral current becomes important when the intrinsic $J_D$ is low (high $E_{CT}$). In the scope of Zheng *et al.*'s work, the authors have used mostly high gap materials, so that this effect was clearly observed. For low $E_{CT}$ BHJs, the intrinsic $J_D$ is much higher than the lateral contribution, such that its effect becomes irrelevant.

Summarizing the optimizations that have been performed, $J_D$ is affected by selectivity of the contacts, blocking layers usage and device structuring. However, the low $E_{CT}$ BHJ TPDP:$C_{60}$ seems to be unaffected by any of these optimization proceedings, including thickness variation.

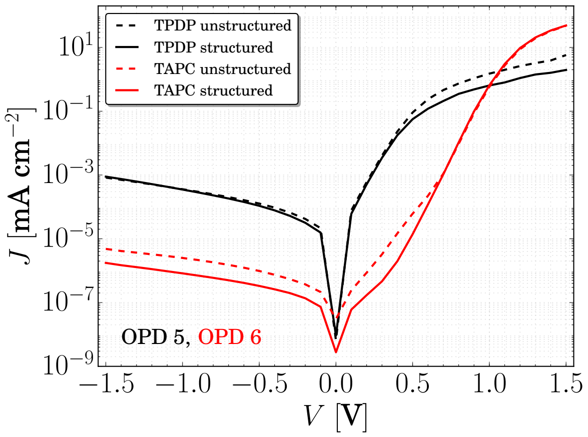


**Supplementary Figure 4: Dark currents and device structuring** | $JV$ curves for devices with TPDP as donor using structured (solid) and unstructured (dashed lines) are drawn in black. Likewise, TAPC is drawn in red. Upon structuring $J_\mathrm{D}$ remains unchanged in reverse bias for TPDP and considerably decrease for TAPC.

Thus, we have strong indications that further effects, than a matter of device optimization, are causing high $J_\mathrm{D}$.

Considering that the BHJ results, discussed in Figure 1 in the main text, are acquired by means of the afore-discussed optimization and, more importantly, rely on the same device structure and material combination, except for the donor blended with $C_{60}$, it is reasonable to assume that the high $J_\mathrm{D}$ is an intrinsic property of each material combination. As argued in the main text, experimental $J_\mathrm{D}$ cannot be explained solely by thermal excitation through CT states.

# 2 Supplementary Note 2. Reconstruction of the Trap Density of States via Impedance Spectroscopy

The trap distribution is determined using the method proposed by Walter *et al.*[9]. In this method, the trap concentration is reconstructed based on its contribution to the device capacitance, when the trap states are filled by an AC signal. The thermal emission of charges from traps states happens with a specific time constant, $\tau_\mathrm{t}$. Therefore, the maximum frequency $f_\mathrm{t}$ at which trapped charges can respond to the applied signal can be determined as $1/\tau_\mathrm{t}$. We can write $f_\mathrm{t}$ in terms of the angular frequency of the AC modulation[10]:

$$\omega_\mathrm{t} = 2\nu_0 \exp\left(-\frac{E_\mathrm{t}}{k_\mathrm{B}T}\right), \quad \text{(S2.1)}$$

where $E_\mathrm{t}$ is the trap energy with respect to the transport energy and $\nu_0$ is the attempt-to-scape frequency[11]. Equation (S2.1) relates the trap energy to the modulation frequency of the signal. Different trap energies can be probed as each trap energy corresponds to a transition in the

$C$-$f$ spectrum. The attempt-to-scape frequency is also related to the thermal velocity, $v_{\text{th}}$, and capture cross-section ($\sigma_{\text{n,p}}$), via[10]:

$$\nu_0 = N_{\text{n,p}} v_{\text{th}} \sigma_{\text{n,p}} = N_{\text{n,p}} \beta_{\text{n,p}}, \tag{S2.2}$$

which serves an approximation for the recombination rate $\beta_{\text{SRH}}$. An exact agreement ($\beta_{\text{SRH}}$ $= \nu_0 N_{\text{n,p}}^{-1}$) is not expected here, as $N_{\text{n,p}}$ is unknown. The trap contribution to the capacitance has been derived based on the Boltzmann occupation of trap states with respect to the Fermi level ($E_{\text{F}}$) and can be written as:

$$C_{\text{t}}' = \frac{q^2}{k_{\text{B}}T} \frac{\tilde{u}_{\text{n,p}}}{\tilde{u}_{\text{ext}}} \int_{-\infty}^{+\infty} \left[ 2 + \exp\left( -\frac{E - E_{\text{F}}}{k_{\text{B}}T} \right) + \exp\left( -\frac{E_{\text{F}} - E}{k_{\text{B}}T} \right) \right]^{-1} N_{\text{t}}(E) dE, \tag{S2.3}$$

where $\tilde{u}_{\text{n,p}}$ and $\tilde{u}_{\text{ext}}$ are the local shift in the quasi-fermi level and the external perturbation, respectively. According to equation (S2.3), when the frequency of the external perturbation is low enough, traps states crossed by the Fermi-level contribute more strongly to the capacitance. As the distance of trap states from $E_{\text{F}}$ increases, their contribution decreases exponentially.

Considering $\tilde{u}_{\text{n,p}}$ and $\tilde{u}_{\text{ext}}$ constant and assuming that the trap distribution is constant in the interval $E_{\text{F}} \pm 2k_{\text{B}}T$, equation (S2.3) can be integrated in the depletion region, from which an analytic expression for $N_{\text{t}}$ can be derived:

$$N_{\text{t}}(E) = -\frac{V_{\text{bi}}}{qW} \frac{\omega}{k_{\text{B}}T} \frac{dC}{d\omega}, \tag{S2.4}$$

where $V_{\text{bi}}$ is the built-in voltage and $W$ is the space charge width region.

# 3 Supplementary Note 3. Impedance spectroscopy in Organic Blends

Using the method introduced by Walter *et al.*[9] to characterize trap states organic devices is debated in literature, especially when dealing with low mobility materials or devices where energy barriers are present. As both conditions apply for our devices, we exemplary compare different devices and analyze the effects on the trap characterization to ensure that the results discussed within this chapter can be accurately estimated. In order to do that, we analyze devices comprising different thicknesses and under different biasing conditions.

In Supplementary Figure 5 data for devices with different active layer thicknesses is shown. The trap density is not expected to depend/vary with thickness. Indeed, from 50 to 150 nm, $N_{\text{t}}$ remains constant. As indicated in the main text, the attempt-to-scape frequency ($\nu_0$) is obtained by overlapping $N_{\text{t}}$ measured at different temperatures. For 150 nm, in order to achieve that, $\nu_0$ has to be set to a lower value. As pointed out by different research groups, thicker devices[10]

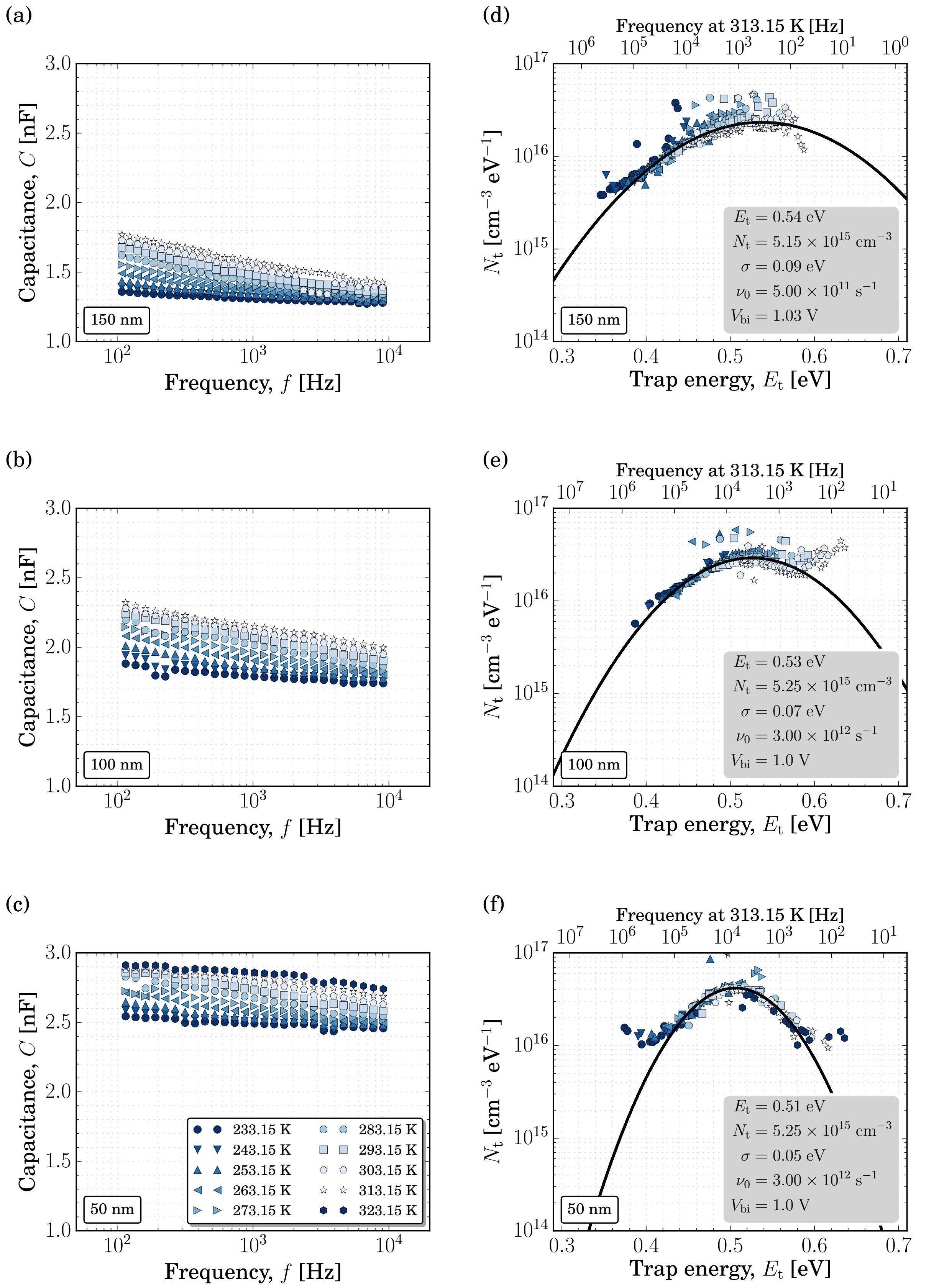


**Supplementary Figure 5: Trap analyzes for TPDP:C$_{60}$ (13.3 mol%) with different device thicknesses (a-c)** Capacitance and **(d-e)** trap density.

and low mobility materials[12] lead to a wrong estimation of $\nu_0$. This further explains why using equation (S2.2) as a direct estimation of the recombination rate is not possible as $\nu_0$ can be underestimated. Therefore, the values of $\nu_0$ must be taken only a first approximation in our study, representing a limitation of the method. Also $E_t$ can be slightly affected, depending on the mobility of the blend. The results shown in Supplementary Figure 5 were measured in devices fabricated in the same batch, but in a different batch than that of the samples presented in the main text, explaining the small deviations in the absolute amount of traps.

Another important aspect when applying this method in devices is the presence of energy barriers, as they can produce the same signature in the capacitance spectra as those produced by traps. As argued by Siebentritt *et al.*, the occupancy of trap states is governed by the crossing of the Fermi level with the trap level, therefore, any trap signature should disappear at high enough forward bias, since the Fermi level will no longer cross the trap level[13]. Following the same reasoning, a minority carrier trap signature should also disappear at high enough reverse bias. Indeed, measuring our device at different biases we can clearly observe this effect: the step in the capacitance spectra, observed at zero bias in the range from 10 Hz to 10 kHz, disappears when both forward and reverse bias are used. From this measurement, shown in Supplementary Figure 6, we can infer that the step in the capacitance arises from traps and, more importantly, that these states are minority carrier traps[13].

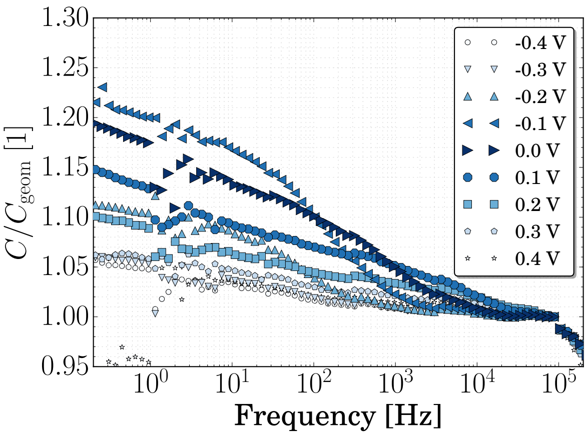


**Supplementary Figure 6: Capacitance spectra at different biases for a device based on TPDP:$C_{60}$ (13.3 mol%)** | Note that the reconstruction of the trap density uses the derivative of the capacitance spectra. This implies that all spectra at bias below -0.3 V, as well as above 0.3 V, lead to the same trap density, which tends to zero, given their rather constant shape.

# 4 Supplementary Note 4. Generation-Recombination Statistics Due to a Distribution of Traps and Drift-Diffusion Modeling

When recombination centers, such as the ones measured in the previous section, are found within the energy gap of semiconductors, they contribute to the generation and recombination processes. This is a consequence of the static of occupation of these states, which happens through the excitation of charges carriers. SRH theory was firstly derived for a single trap level and is based on four rates of capture (energy absorption) and release (energy emission) of charges [14], as schematically represented in Supplementary Figure 7.

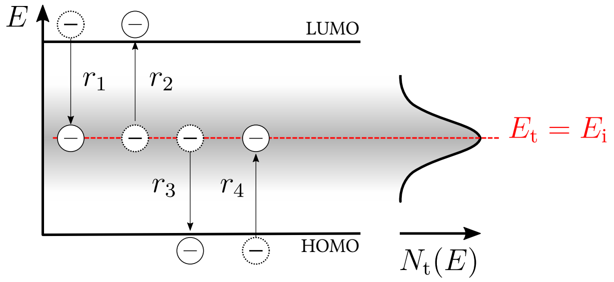


**Supplementary Figure 7: Rates of capture and release of charges in the SRH framework**. $r_1$ and $r_2$ represents capture and emission of electrons, respectively. $r_3$ and $r_4$ are the equivalent for holes. $E_\mathrm{t}$ represents a discrete trap energy in the trap distribution $N_\mathrm{t}$. $E_\mathrm{i}$ is the mid-gap energy.

In order to obtain the rates shown in Supplementary Figure 7, one assumes a Fermi-Dirac occupation function $f(E,T)$ for the probability that a trap at energy $E_\mathrm{t}$ is occupied. The rates can then be written as [15]:

$$\begin{aligned} r_1 &= n v_\mathrm{th} \sigma_\mathrm{n} N_\mathrm{t}[1 - f(E,T)] && \text{Electron capture} \\ r_2 &= e_\mathrm{n} N_\mathrm{t} f(E,T) && \text{Electron emission} \\ r_3 &= p v_\mathrm{th} \sigma_\mathrm{p} N_\mathrm{t} f(E,T) && \text{Hole capture} \\ r_4 &= p v_\mathrm{th} \sigma_\mathrm{p} N_\mathrm{t} f(E,T) && \text{Hole emission} \end{aligned} \tag{S4.5}$$

In equation (S4.5), $n$ and $p$ are the electron and hole concentration, respectively. $\sigma_\mathrm{n,p}$ and $e_\mathrm{n,p}$ are the capture cross-section and emission coefficients, respectively, for holes (p) and electrons (e). $v_\mathrm{n,p}$ is the thermal velocity. The product $\sigma_\mathrm{n,p} v_\mathrm{n,p}$ can be estimated from the trap analyses via equation (S2.2). Because $N_\mathrm{n,p}$ are unknown, a direct input via equation (S2.2) was not considered and the value was adjusted to achieve a good agreement with the experimental curve.

In thermal equilibrium, the capture and emission rates for electrons and holes must be equal ($r_1 = r_2$, $r_3 = r_4$). With this assumption, using the set of equations above, $e_\mathrm{n}$ and $e_\mathrm{n}$ can be found. We define the net generation/recombination efficiency as:

$$\eta_\mathrm{G,R} = r_1 - r_2 = r_3 - r_4. \tag{S4.6}$$

Once more using the set of equation above and equation (S4.6), we can derive the occupation function (solving equation (S4.6) for $f(E,T)$) and generation/recombination rate, which can be written as:

$$\eta_{\mathrm{G,R}} = v_{\mathrm{th}}\sigma_{\mathrm{n}}\sigma_{\mathrm{p}}\frac{np - n_{\mathrm{i}}^2}{nv_{\mathrm{th}}\sigma_{\mathrm{n}} + pv_{\mathrm{th}}\sigma_{\mathrm{p}} + e_{\mathrm{n}} + e_{\mathrm{p}}}. \tag{S4.7}$$

By multiplying equation (S4.7) by the number of trap states ($N_{\mathrm{t}}$) we can obtain the rate of generation ($G$) and recombination ($R$), which are related as $G = -R$. We are interested in the reverse region of the $JV$ curve, because photodetectors are mainly operated at negative bias. Considering that, we can make some approximations to understand the behavior of equation (S4.7). Firstly, under reverse conditions, the concentration of free charges in the device is very small, because they are easily extracted by the drift-applied field. Moreover, the capture rates are proportional to the number of free charges, making these processes irrelevant for the device. This also allows considering $\sigma_{\mathrm{n}} = \sigma_{\mathrm{p}} = \sigma_0$.

In reverse bias, the important processes are the emission of electron and holes assisted by the trap states. These electrons (holes) are then emitted to the EA (IP), increasing the dark current value. Applying the aforementioned considerations/assumptions and expressing $e_{\mathrm{n}}$ and $e_{\mathrm{n}}$ in terms of appropriated variables, equation (S4.7) can be written as Equation (4) in the main text:

$$G_{\mathrm{SRH}} = \frac{\sigma_0 v_{\mathrm{th}} N_{\mathrm{t}}}{2\cosh\left(\frac{E_{\mathrm{t}} - E_{\mathrm{i}}}{k_{\mathrm{B}}T}\right)} n_{\mathrm{i}} \tag{4}$$

Equation (4) has its maximum value when $E_{\mathrm{t}} = E_{\mathrm{i}}$. This means that trap states close to mid gap are the most relevant for generation.

Recalling the experimental results presented in the main text, in the trap distribution, $E_{\mathrm{t}}$ values cross or are very close to mid gap, explaining the high values of $J_{\mathrm{D}}$ as well as its increase with the number of traps. Since the devices measured presented a distribution of traps that could be approximated by a Gaussian distribution, we model the SRH generation in the drift-diffusion simulation by integrating the product of equation (S4.7) by the measured distribution over the entire band gap:

$$G = \int_{\mathrm{HOMO_D}}^{\mathrm{LUMO_A}} N_{\mathrm{t}}(E)\eta_{G,R} dE. \tag{S4.8}$$

# 5 Supplementary Note 5. Noise Measurements

The setup is shown in Supplementary Figure 8a. The circuit consists of an input stage transimpedance amplifier that converts the current through the OPD into the voltage $v_{o1}$, followed by two stages of high-pass filter (HPF) and two stages of signal amplification (gain) plus low-pass filter (LPF). The output signal $v_{out}$ is then sampled at 4 to 12 million points in real-time using an oscilloscope with 16-bit of resolution. The spectrum of $v_{out}$ is calculated using the Welch's method for estimating the power spectral density ($S_n$) in MATLAB[16]. The LPFs and HPFs significantly attenuate the signal power content outside of the target frequency bandwidth. This prevents any mistranslation of non-target signal power into the target measurement bandwidth.

Since the transfer function of each stage and the noise of intermediate components are known, $S_n$ of $v_{o1}$ can be calculated back from $v_{out}$. Then the total noise current ($i_{total}$) would be $v_{o1}$ divided by $R_1||C_1$ impedance. $i_{total}$ has several known sub-components that can be removed by subtracting their power to extract the net OPD noise. The noise voltage and noise current of the amplifiers[1] are known from the datasheet or separate measurements. These sub-components could dominate the OPD noise at very low or very high frequencies and therefore limit the frequency range that the OPD noise can be accurately extracted.

Very low noise operational amplifiers are used in the circuit; $O_1$ and $O_2$ are CMOS amplifiers with an input bias current of $\simeq 1$ pA and a gain bandwidth product of 28 MHz. $O_2$ and $O_3$ are bipolar-junction-transistor (BTJ) amplifiers with a gain bandwidth product of 200 MHz. On-board batteries are used for biasing the OPD and powering the circuit. Especially, Nickel-Cadmium batteries with $< 100\,\mathrm{m\Omega}$ series resistance are needed for both positive and negative supply rails of the op-amps to eliminate any feedback from outputs of $O_3$, $O_4$ and $O_5$ to the $O_1$ through supply rails. Therefore, small low-noise resistors are needed in the feedback loops of $O_3$ and $O_4$ that draw high current from the supply rails. For the same reason, the oscilloscope is used in the high impedance input mode.

[1] AD8656, LT6236, and LTC6240, Analog Devices Inc., USA

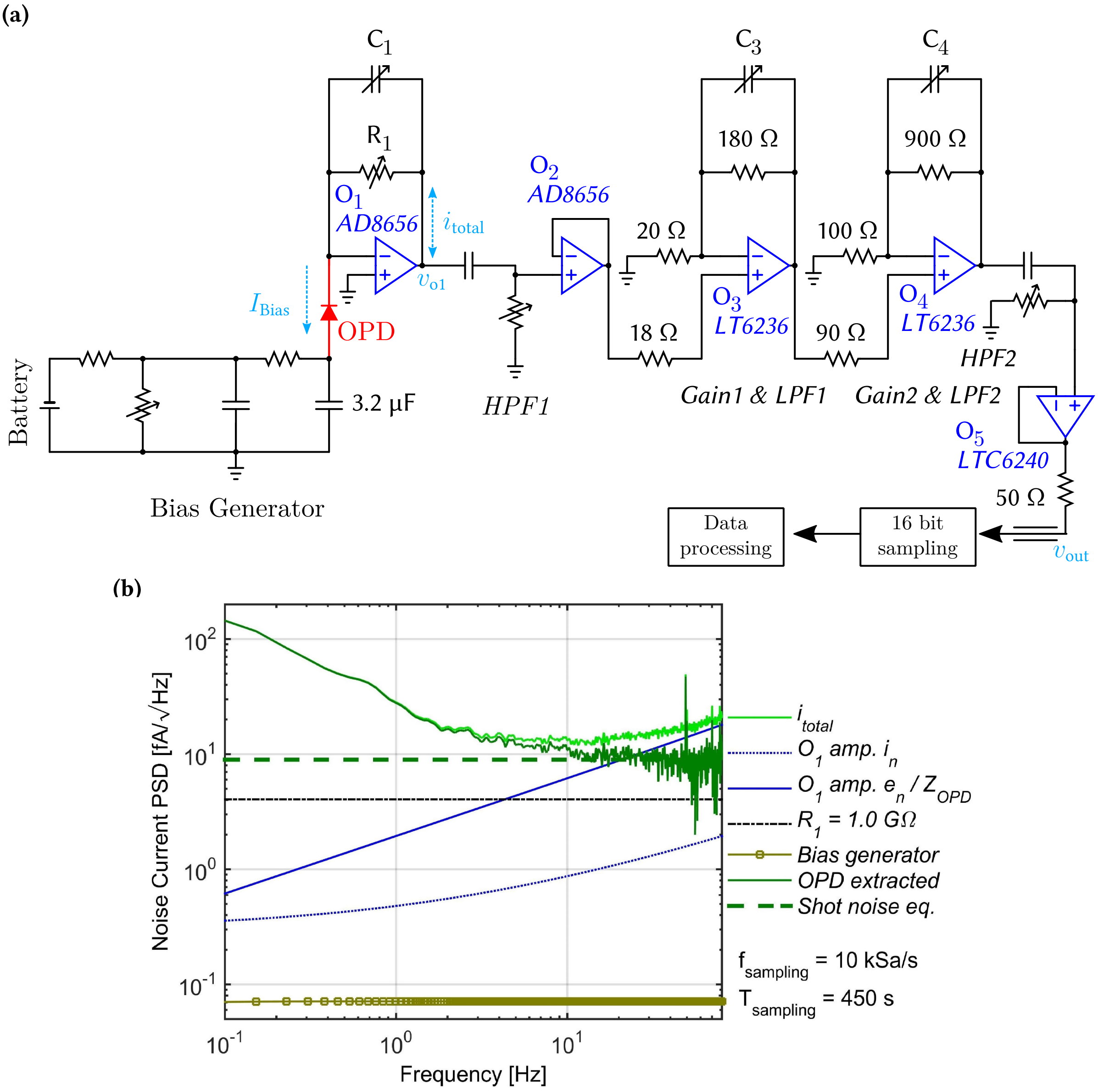


**Supplementary Figure 8: Noise measurement** | **(a)** Simplified schematic representation of the noise measurement setup. **(b)** An example of noise extraction for Spiro-MeO-TPD:$C_{60}$ BHJ at $I_{\text{bias}} = 250\,\text{pA}$ reverse bias.

# 6 Supplementary Tables

**Supplementary Table 2: Donors used to fabricate different photodiodes** | $E_{CT}$ varies from 0.85 to 1.58 eV when blended to $C_{60}$ at 6 mol%. The CT absorption tails in Supplementary Figure 9 were fitted as described elsewhere[17]. From fitting the $EQE$ spectra, values of $E_{CT}$, $\lambda_{CT}$ and $f_{CT}$ were extracted, as listed here. The statistical error of the fitting procedure is estimated by systematically changing the start and end value of the fit, resulting to 25 different fits.

| **N°** | **Short Name**<br>*Long Name*<br>*(Supplier)* | **Chemical structure** | $E_{CT}$ **[meV]** | $\lambda_{CT}$ **[meV]** | $f_{CT}$ **[µ(eV)$^2$]** |
|---|---|---|---|---|---|
| 1 | **TTDTP**<br>*2,2',6,6'-tetra-p-tolyl-4,4'-bithiopyranylidene*<br>*(TU Dresden, Germany)* | 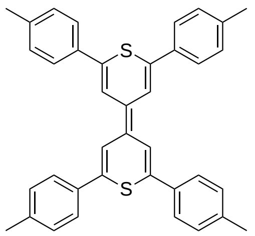 | 851 ± 1 | 172 ± 4 | 6.7 ± 0.3 |
| 2 | **TPDP**<br>*2,2',6,6'- tetraphenyl-4,4'- bipyranylidene*<br>*(TU Dresden, Germany)* | 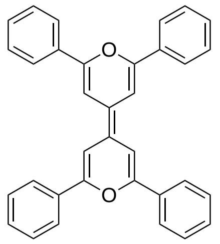 | 903 ± 1 | 178 ± 6 | 9.4 ± 0.6 |
| 3 | **m-MTDATA**<br>*4,4',4"-tris(3-m-tolyl-phenylamino)-triphenylamine*<br>*(Luminescence Technology Corp., Taiwan.)* | 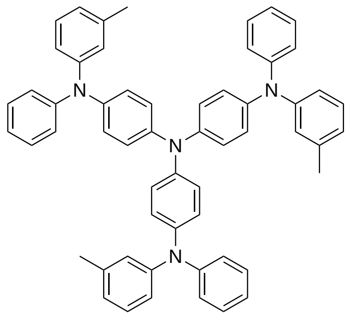 | 1029 ± 4 | 453 ± 8 | 148.9 ± 3.5 |

*Continued on next page*

Supplementary Table 2 – *Donors used to fabricate different photodiodes.*

| N° | **Short Name**<br>*Long Name*<br>*(Supplier)* | **Chemical structure** | $E_{CT}$ **[meV]** | $\lambda_{CT}$ **[meV]** | $f_{CT}$ **[μ(eV)$^2$]** |
|---|---|---|---|---|---|
| 4 | **Spiro-MeO-TPD**<br>*2,7-bis[N,N-bis(4-methoxy-phenyl)amino] 9,9-spiro-bifluorene*<br>*(Luminescence Technology Corp., Taiwan.)* | 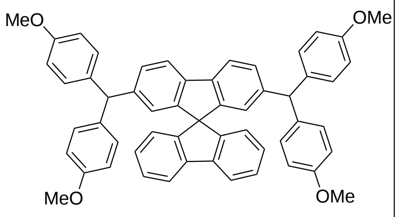 | 1106 ± 1 | 207 ± 6 | 59.4 ± 4.3 |
| 5 | **ZnPc**<br>*zinc-phthalocyanine*<br>*(CreaPhys GmbH)* | 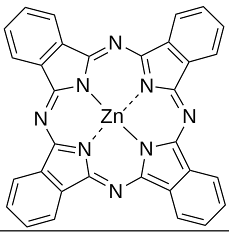 | 1195 ± 1 | 425 ± 3 | 2037.8 ± 53.5 |
| 6 | **TAPC**<br>*1,1-bis[4-(N,N-di-p-tolylamino)phenyl] cyclohexane*<br>*(Luminescence Technology Corp., Taiwan.)* | 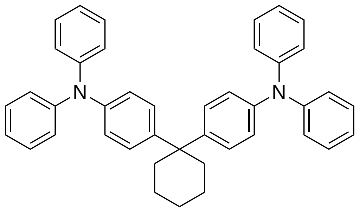 | 1426 ± 1 | 196 ± 11 | 709.4 ± 77.6 |
| 7 | **P4-Ph4-DIP**<br>*2,3,10,11-tetrapropyl-1,4,9,12-tetraphenyl-diindeno[1,2,3-cd:1',2',3'-lm]perylene*<br>*(TU Dresden, Germany)* | 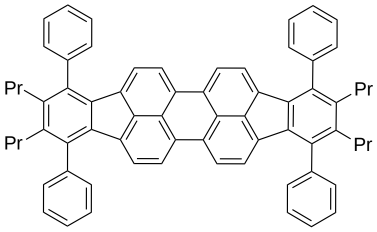 | 1585 ± 3 | 159 ± 18 | 1272.4 ± 210.3 |

**Supplementary Table 3: Materials used to fabricate the devices** | HTL and ETL stands for hole transport layer and electron transport layer, respectively. Likewise, EBL and HBL stands for electron blocking layer and hole blocking layer, respectively.

| N° | **Short Name**<br>*Long Name*<br>*(Supplier)* | **Chemical structure** | **Used as** |
|---|---|---|---|
| 1 | **$C_{60}$**<br>*Buckminsterfullerene*<br>*(Luminescence Technology Corp., Taiwan.)* | 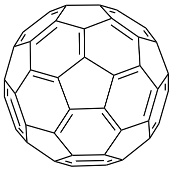 | Acceptor |
| 2 | **MeO-TPD**<br>*N,N,N',N'-Tetrakis (4-methoxyphenyl)-benzidine*<br>*(Luminescence Technology Corp., Taiwan.)* | 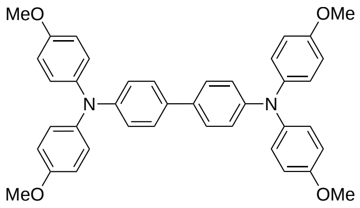 | HTL/EBL |
| 3 | **HATNA-$Cl_6$**<br>*2,3,8,9,14,15-hexachloro-5,6,11,12,17,18-hexaazatrinaphthylene*<br>*(Luminescence Technology Corp., Taiwan.)* | 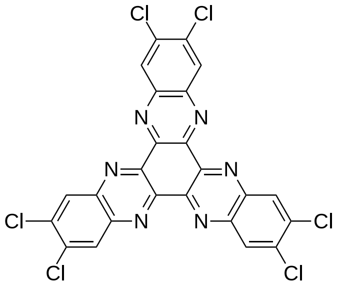 | ETL/HBL |
| 4 | **HAT(CN)$_6$**<br>*hexaazatriphenylene-hexacarbonitril*<br>*(Luminescence Technology Corp., Taiwan.)* | 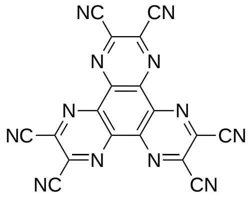 | ETL/HBL |

*Continued on next page*

Supplementary Table 3 – *Materials used to fabricate the devices.*

| № | **Short Name** *Long Name* *(Supplier)* | **Chemical structure** | **Used as** |
|---|---|---|---|
| 5 | **BPhen** *Bathophenanthroline* *(Luminescence Technology Corp., Taiwan.)* | 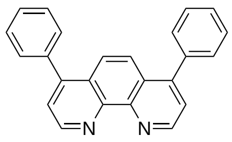 | ETL |
| 6 | **$F_6$-TCNNQ** *2,2'-(perfluoronaphthalene-2,6-diylidene) dimalononitrile* *(Novaled GmbH)* | 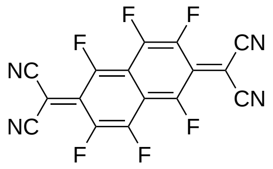 | p-dopant |
| 7 | **$W_2$(hpp)$_4$** *tetrakis(1,3,4,6,7,8-hexahydro- 2H-pyrimido [1,2-a] pyrimidinato) ditungsten (II)* *(Novaled GmbH)* | 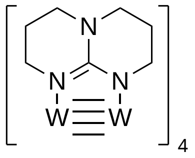 | n-dopant |

**Supplementary Table 4: Ideal value of the saturation current** | $J_0$ was calculated using Equation (1) and (2) of the main text.. The parameters used in the calculation were extracted from Supplementary Table 2 and $EQE_{\mathrm{EL}}$ was estimated following Ref.[17] according to the voltage losses analyses. For ZnPc:$C_{60}$ (50 wt%), $f_{\mathrm{CT}}$ and $\lambda_{\mathrm{CT}}$ are 288.0 meV and 4.0 m(eV)$^2$, respectively.

| Donor molecule | $E_{\mathrm{CT}}$ [eV] | $V_{\mathrm{OC}}$ [mV] | $\Delta V_{\mathrm{rec}}$ [mV] | $\Delta V_{\mathrm{rad}}$ [mV] | $\Delta V_{\mathrm{nonrad}}$ [mV] | $EQE_{\mathrm{EL}}$ [1] | $J_0$ [mA cm$^{-2}$] |
|---|---|---|---|---|---|---|---|
| TTDPT | 0.85 | 307 | 543 | 124 | 419 | $6 \times 10^{-8}$ | $4 \times 10^{-6}$ |
| TPDP | 0.90 | 309 | 591 | 137 | 454 | $2 \times 10^{-8}$ | $3 \times 10^{-6}$ |
| m-MTDATA | 1.04 | 434 | 596 | 181 | 415 | $8 \times 10^{-8}$ | $4 \times 10^{-8}$ |
| Spiro-MeO-TPD | 1.10 | 496 | 614 | 223 | 391 | $2 \times 10^{-7}$ | $5 \times 10^{-10}$ |
| ZnPc | 1.19 | 583 | 607 | 278 | 329 | $2 \times 10^{-6}$ | $4 \times 10^{-11}$ |
| TAPC | 1.42 | 836 | 594 | 257 | 337 | $3 \times 10^{-6}$ | $3 \times 10^{-15}$ |
| P4-Ph4-DIP | 1.58 | 949 | 631 | 344 | 287 | $1 \times 10^{-5}$ | $3 \times 10^{-18}$ |
| ZnPc (50 wt%) | 1.15 | 555 | 595 | 220 | 375 | $4 \times 10^{-7}$ | $3 \times 10^{-9}$ |

**Supplementary Table 5: CT state properties of TPDP:$C_{60}$ devices extracted from fits in Supplementary Figure 10.**

| Concentration [mol%] | $E_{CT}$ [meV] | $\lambda_{CT}$ [meV] | $f_{CT}$ [$\mu$(eV)$^2$] |
|---|---|---|---|
| 6.0 | $904 \pm 1$ | $139 \pm 9$ | $1.7 \pm 0.2$ |
| 10.5 | $900 \pm 1$ | $147 \pm 11$ | $2.3 \pm 0.3$ |
| 13.3 | $897 \pm 1$ | $154 \pm 10$ | $2.0 \pm 0.2$ |
| 16.1 | $895 \pm 2$ | $162 \pm 11$ | $1.7 \pm 0.2$ |
| 18.8 | $898 \pm 2$ | $155 \pm 12$ | $1.6 \pm 0.2$ |
| 21.5 | $897 \pm 2$ | $160 \pm 12$ | $1.4 \pm 0.2$ |
| 24.1 | $890 \pm 1$ | $179 \pm 9$ | $1.1 \pm 0.1$ |
| 26.7 | $887 \pm 1$ | $186 \pm 9$ | $1.1 \pm 0.1$ |

**Supplementary Table 6: Parameters used in the drift-diffusion simulation showed in Figure 4a,b and Figure 5b in the main text.**

| | ZnPc:$C_{60}$ | TPDP:$C_{60}$ | Unit |
|---|---|---|---|
| Trap distribution | As measured (see Supplementary Figure 17) | As measured (see Supplementary Figure 11) | See parameters |
| ${\beta_{SRH}}^a = v_{n,p}\sigma_{n,p} = \nu_0 N_{n,p}^{-1}$ | $1 \times 10^{-17}$ | $2 \times 10^{-17}$ | $m^3\,s^{-1}$ |
| $\beta_{bimolecular}$ | $5 \times 10^{-18}$ | $5 \times 10^{-19}$ | $m^3\,s^{-1}$ |
| Effective thickness$^b$ | $50 + 50 + 50$ | $50 + 50 + 50$ | nm |
| $\mu_{n,p}$ | $9 \times 10^{-9}$ | $2 \times 10^{-9}$ | $m^2\,V^{-1}\,s^{-1}$ |
| $\epsilon$ | 5 | 5 | |
| $V_{bi} = \phi_{cathode} - \phi_{anode}$ | 0.97 | 0.60 | V |

[a] See discussion about the accuracy of this approximation in section 3.

[b] EBL + active layer + HBL. The simulation is performed for a single-layer device with ohmic contacts and an effective thickness, which accounts for the field drop along undoped EBL, active layer and HBL. The generation is limited within the active layer. As suggested by the experimental data, traps are found only in this region supporting this approach.

# 7 Supplementary Figures

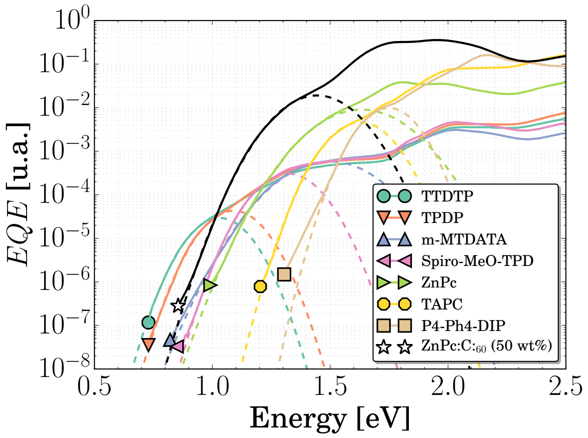


**Supplementary Figure 9: charge-transfer (CT) state properties of donor:C$_{60}$** | **(a)** Sensitively measured $EQE$ spectra (solid line) and the corresponding fits of the CT state absorption (dashed line) for the donor:C$_{60}$ BHJs. The black curve represents ZnPc:C$_{60}$ (50 wt%).

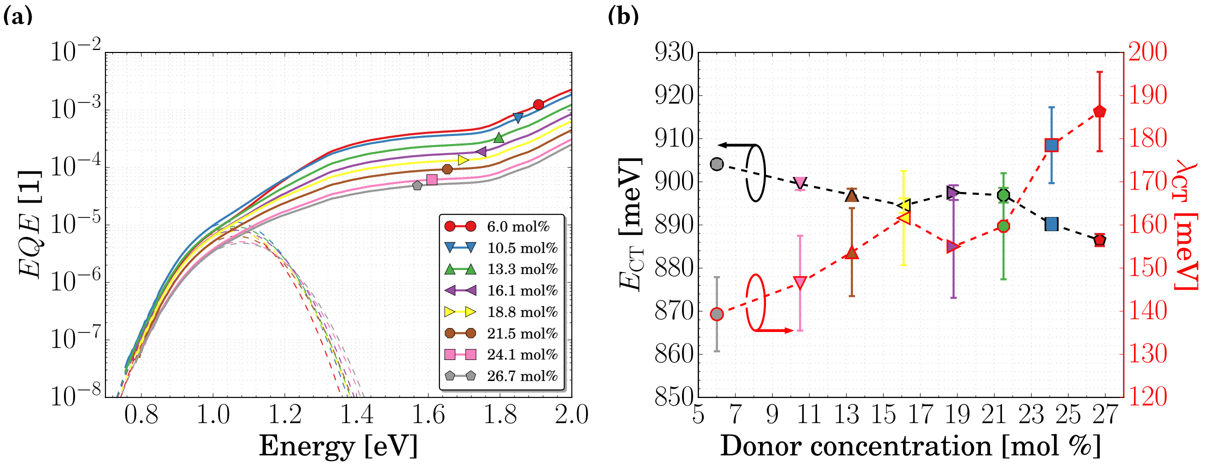


**Supplementary Figure 10: CT state properties in TPDP:C$_{60}$ devices at different concentrations** | **(a)** Sensitively measured $EQE$. **(b)** $E_{CT}$ and $\lambda_{CT}$ extracted from **(a)**. Note that $E_{CT}$ increases only about 20 meV when the concentration varies from 6.0 mol% to 26.7 mol%. The statistical error of the fitting procedure is estimated by systematically changing the start and end value of the fit, resulting to 36 different fits.

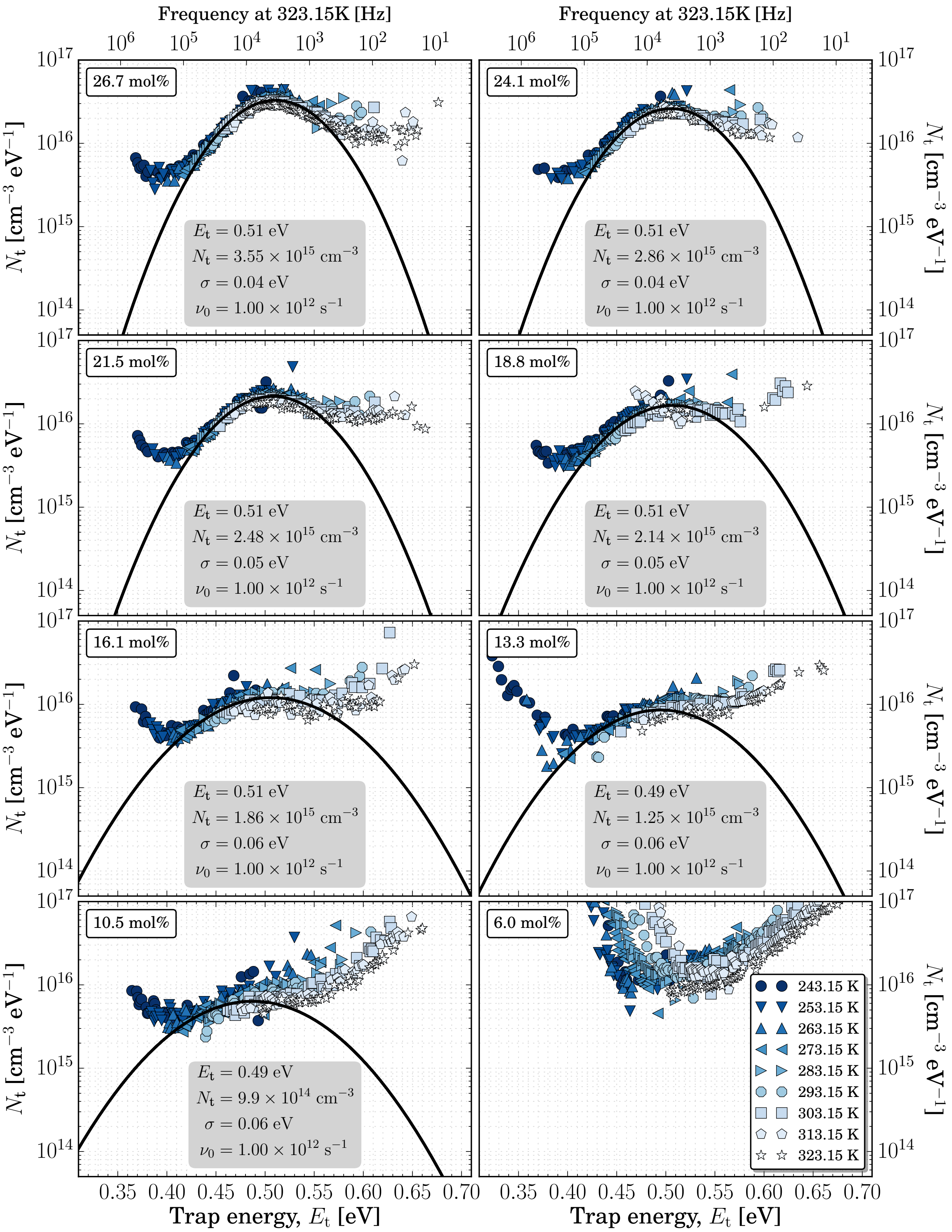


**Supplementary Figure 11: Trap analyses for TPDP:$C_{60}$ at different concentrations** | Trap concentration measured for all devices comprising TPDP:$C_{60}$ as active layer. For 6.0 mol% a fitting was not achieved and no parameters could be extracted. In equation (S2.4), $V_{bi}$ is assumed to be 1.0 V, calculated according to Mantri *et al.*[18].

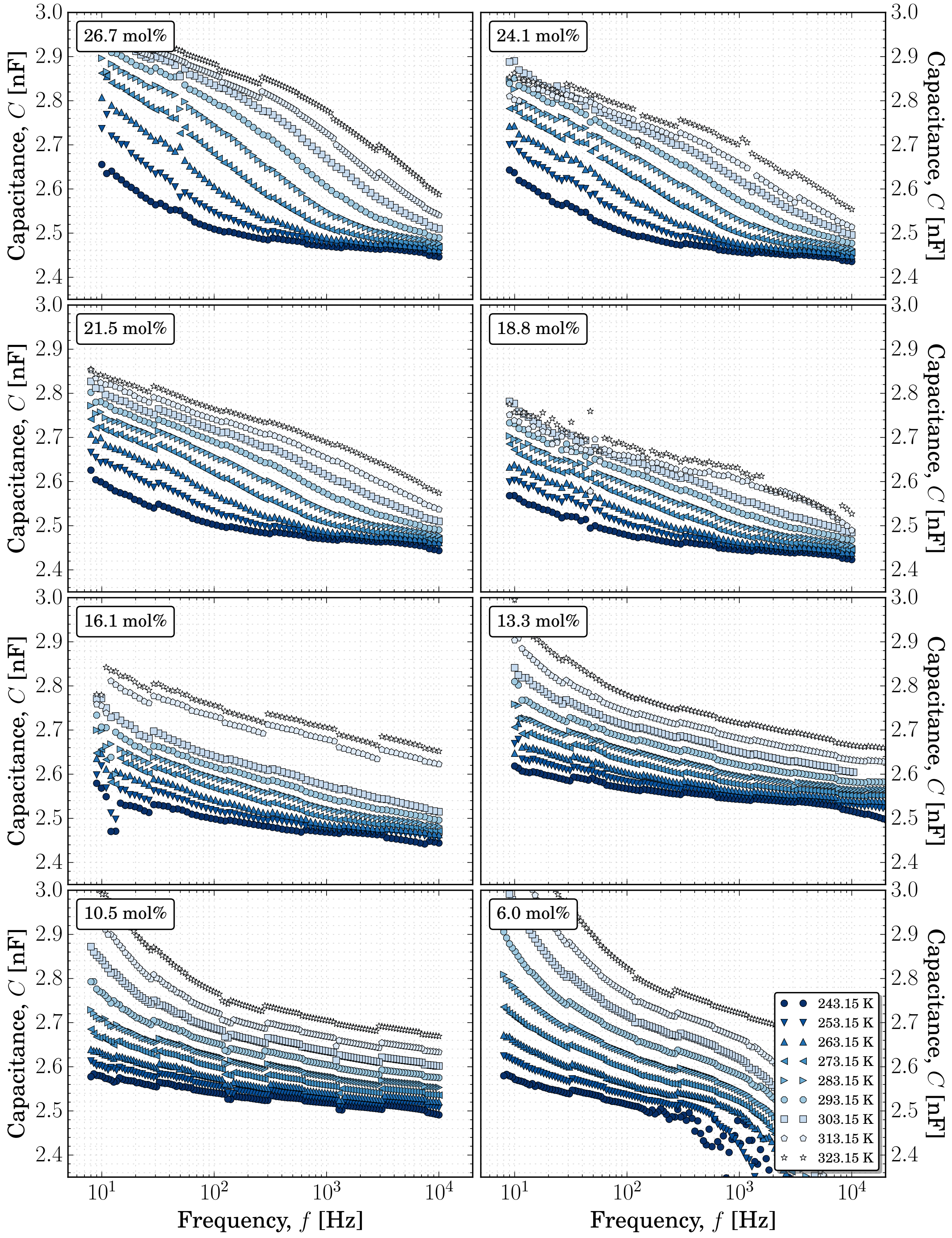


**Supplementary Figure 12: Capacitance for TPDP:$C_{60}$ at different concentration.**

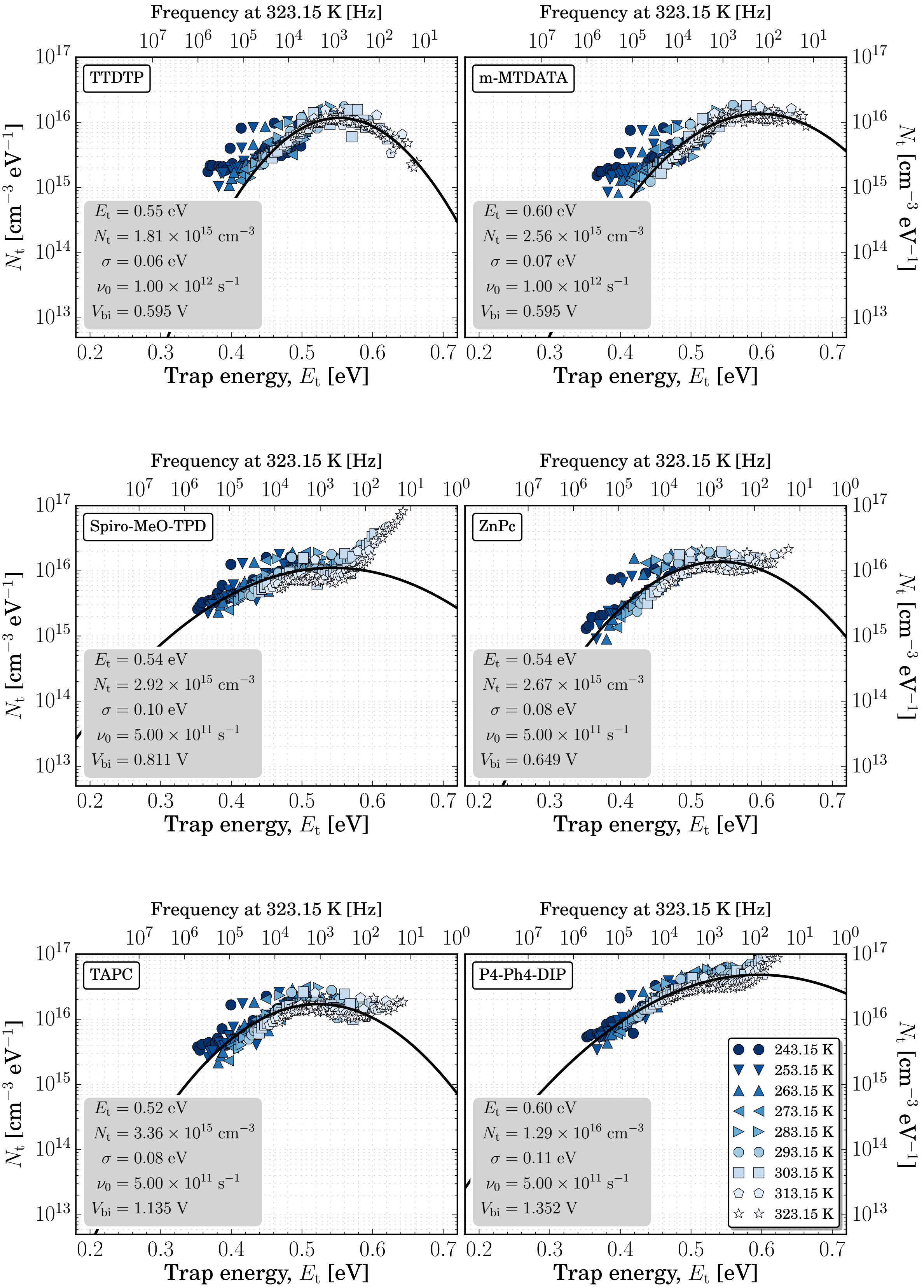

Frequency at 323.15 K [Hz]
Trap energy, $E_t$ [eV]
$N_t$ [cm$^{-3}$ eV$^{-1}$]
TTDTP
$E_t = 0.55$ eV
$N_t = 1.81 \times 10^{15}$ cm$^{-3}$
$\sigma = 0.06$ eV
$\nu_0 = 1.00 \times 10^{12}$ s$^{-1}$
$V_{bi} = 0.595$ V
m-MTDATA
$E_t = 0.60$ eV
$N_t = 2.56 \times 10^{15}$ cm$^{-3}$
$\sigma = 0.07$ eV
$\nu_0 = 1.00 \times 10^{12}$ s$^{-1}$
$V_{bi} = 0.595$ V
Spiro-MeO-TPD
$E_t = 0.54$ eV
$N_t = 2.92 \times 10^{15}$ cm$^{-3}$
$\sigma = 0.10$ eV
$\nu_0 = 5.00 \times 10^{11}$ s$^{-1}$
$V_{bi} = 0.811$ V
ZnPc
$E_t = 0.54$ eV
$N_t = 2.67 \times 10^{15}$ cm$^{-3}$
$\sigma = 0.08$ eV
$\nu_0 = 5.00 \times 10^{11}$ s$^{-1}$
$V_{bi} = 0.649$ V
TAPC
$E_t = 0.52$ eV
$N_t = 3.36 \times 10^{15}$ cm$^{-3}$
$\sigma = 0.08$ eV
$\nu_0 = 5.00 \times 10^{11}$ s$^{-1}$
$V_{bi} = 1.135$ V
P4-Ph4-DIP
$E_t = 0.60$ eV
$N_t = 1.29 \times 10^{16}$ cm$^{-3}$
$\sigma = 0.11$ eV
$\nu_0 = 5.00 \times 10^{11}$ s$^{-1}$
$V_{bi} = 1.352$ V
243.15 K
253.15 K
263.15 K
273.15 K
283.15 K
293.15 K
303.15 K
313.15 K
323.15 K

**Supplementary Figure 13 *(previous page)*: Trap analyses for donor:$C_{60}$ (6 mol%) devices**| Trap concentration of donor:$C_{60}$ devices. The data of TPDP:$C_{60}$ is shown in Supplementary Figure 11. For $N_t$ determination with equation (S2.4), $V_{bi}$ was extracted according to Mantri *et al.*[18]. In organic devices, the voltage drops along the organic intrinsic layers. Therefore, the depletion region width ($W$) is assumed to be 90 nm, which corresponds to the device thickness minus the doped layer thicknesses. According to equation (S2.4), variations in $V_{bi}$ and $W$ lead to minor errors in $N_t$, which does not affect the main discussion of this work. As discussed in the main text, the trap density is studied in the region from 10 Hz to 10 kHz, where the device resistance does not play a major role and series resistance can also be neglected. For guidance, the frequency at 323.15 K is also plotted. Note that this frequency corresponds only to one temperature. The fitting range is further adjusted, depending on the material system and the effect of the blend resistance of that specific material system. For Spiro-MeO-TPD and P4-Ph4-DIP, for example, it seems that another type of distribution starts to appear at higher energy. We, however, neglect it as within the frequency range where a reliable result can be obtained and the temperature range we studied, these features could not be properly resolved.

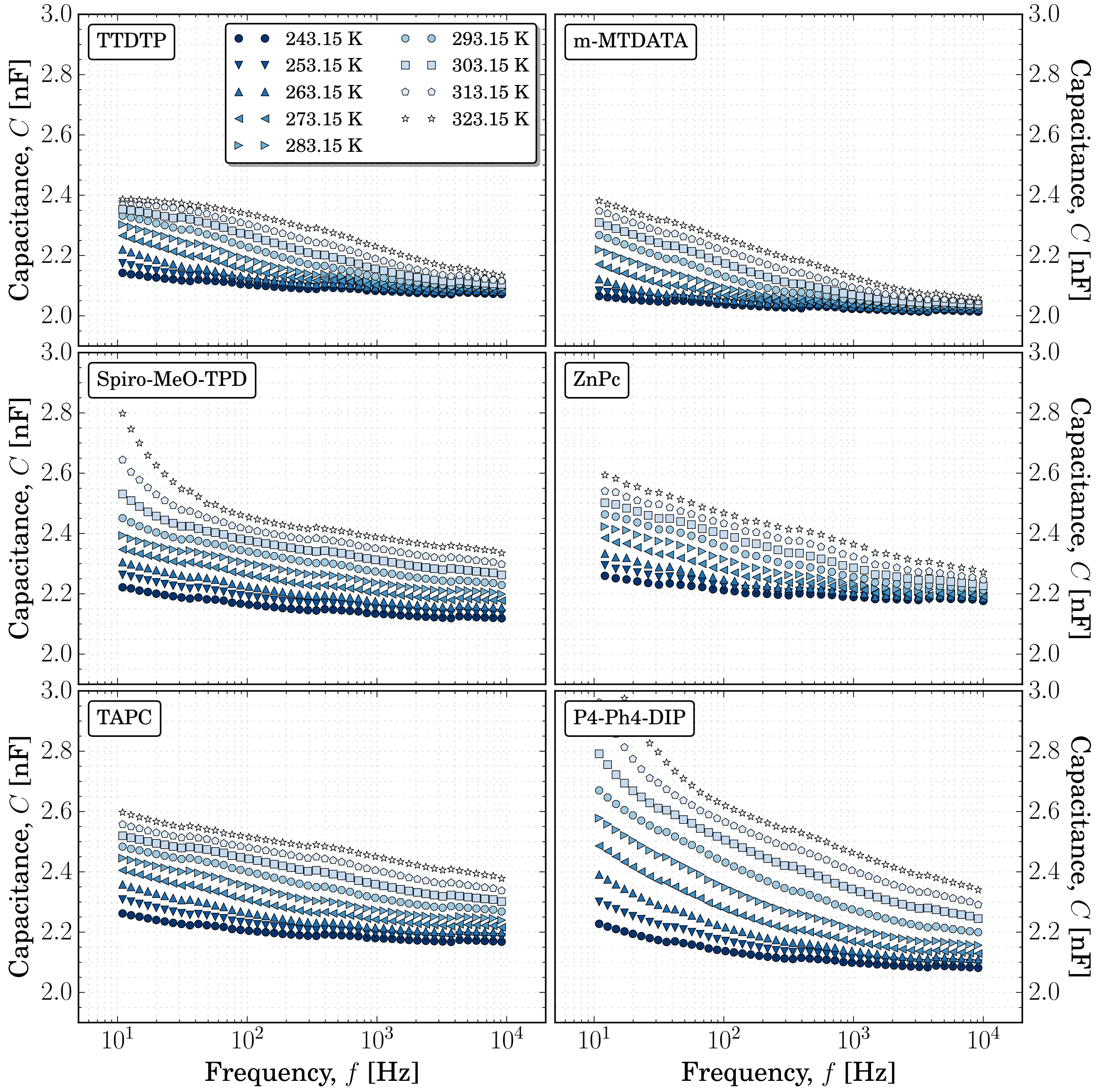


**Supplementary Figure 14: Capacitance for donor:$C_{60}$ (6 mol%) devices** | The data of TPDP:$C_{60}$ is shown in Supplementary Figure 12.

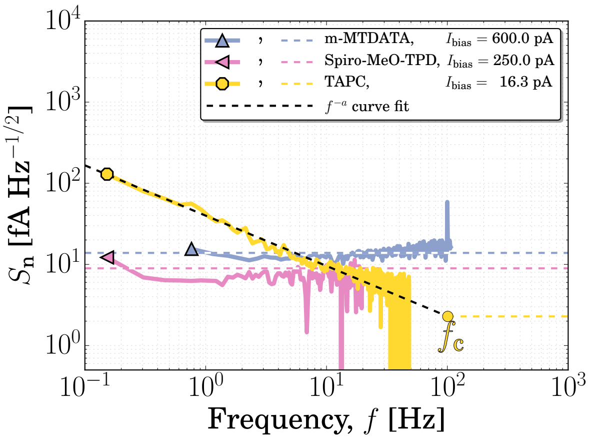


**Supplementary Figure 15: Spectral noise density of three additional donors used in the main text** | Black dashed lines show the fit according to $i_n = \sqrt{2qI_{bias}}$. The noise corner frequency, $f_c$, is determined from the intersection of the shot and $1/f$ component.

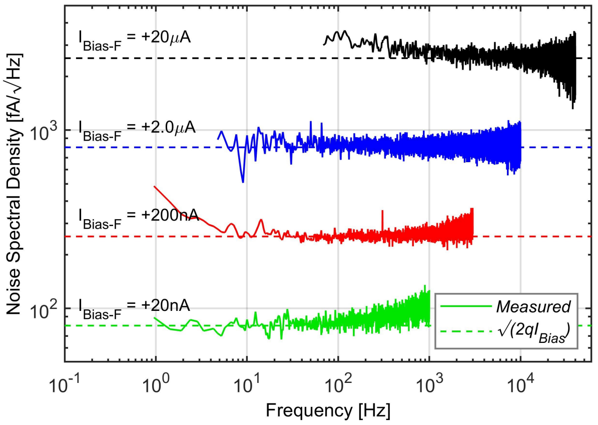


**Supplementary Figure 16: Noise in forward bias.** | TPDP:$C_{60}$ BHJ at different forward bias currents. Dashed lines represent the shot noise calculated as $i_n = \sqrt{2qI_{bias}}$. Here we can clearly see that the $1/f$ noise is still minor even at µA current levels and the measured noise matches very well to the expected theoretical shot noise equation.

**(a)** **(b)**

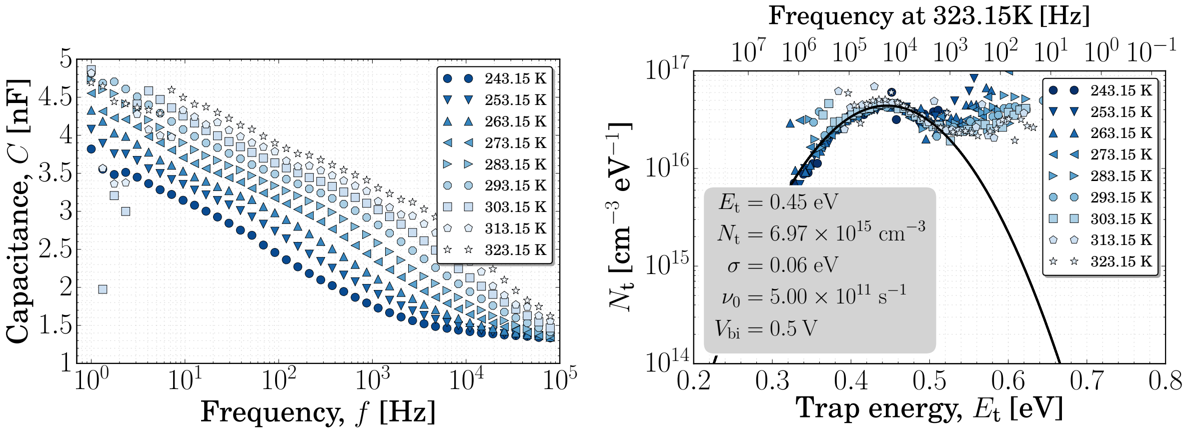


**Supplementary Figure 17: Trap analyzes for ZnPc:$C_{60}$ (50 wt%)** | **(a)** $C$-$f$ spectra and **(b)** Trap density measured at different temperatures.

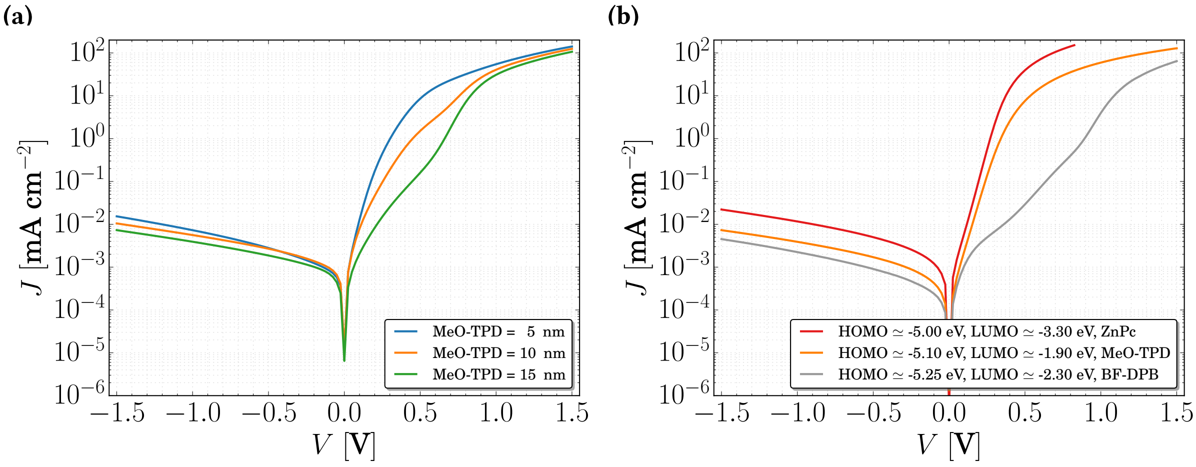


**Supplementary Figure 18: Effects of extraction energy barrier on TPDP:$C_{60}$ devices | (a)** different EBLs are tested with a thickness of 5 nm to study the effect of the HOMO level on $JV$ curves. $J_\mathrm{D}$ is affected by the HOMO level of the HTL/EBL. As the HOMO decreases from -5.0 eV to -5.25 eV, the forward region is increasingly S-kinked. The extraction barrier also affects the reverse region. By increasing the barrier height by ≈250 meV, when comparing ZnPc (HOMO = -5.0 eV)[19] to BF-DPB (-5.25 eV)[20], a decrease of $J_\mathrm{D}$ by one order of magnitude is observed. This suggests that $J_\mathrm{D}$ of TPDP:C60 devices presented in the main text, which use MeO-TPD (HOMO = -5.10 eV)[21], could be higher in the absence of barriers. However, the scaling of $J_\mathrm{D}$ with the trap concentration remains valid, as the barrier is kept constant. Moreover, the LUMO of the EBL/HTL can also affect $J_\mathrm{D}$, as it represent the injection barrier for electrons under reverse bias, which should be as high as possible. **(b)** the thickness of the EBL also affects the forward region of the device. Because this layer is not doped, the field is expected to drop across the layer, which reduces the current when the thickness of the layer increases increase.

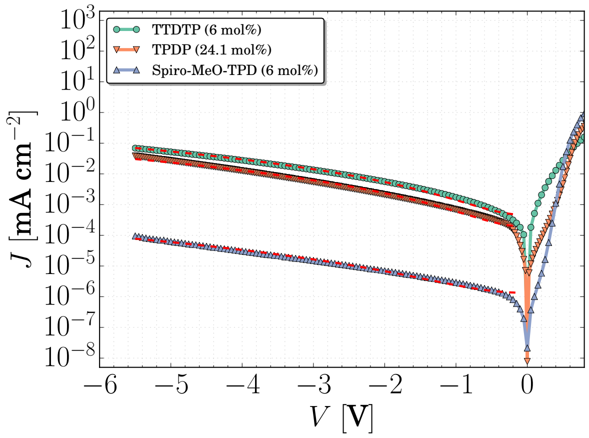


**Supplementary Figure 19: $JV$ characteristics of three different devices |** The devices show an increase in reverse dark current which can be fitted with the analytical solution proposed by Murgatroyd *et al.*[22] showing a dependence on the square root of the applied field. This is a further indication that Poole-Frenkel effect is present in these systems.

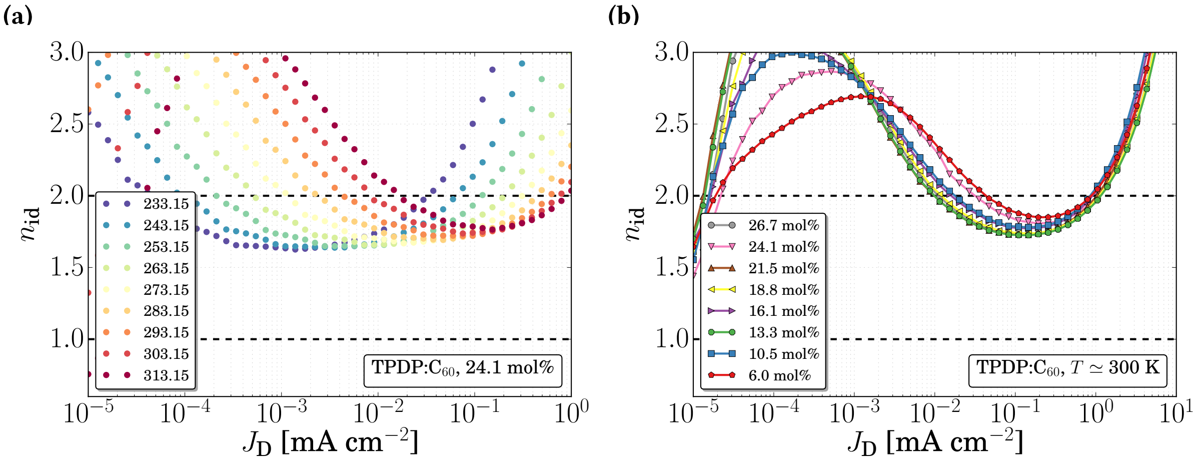


**Supplementary Figure 20: Ideality factor, $n_{\mathrm{id}}$, for TPDP devices | (a)** for 24.1 mol% at different temperatures. A common value of $n_{\mathrm{id}}$ is found for all temperatures, namely around 1.8, in agreement with trap assisted recombination process. At room temperature, $n_{\mathrm{id}}$ can be analyzed at around $10^{-1}$ mA cm$^2$. This is done for all concentration in **(b)**. For these devices, however, we could not observe any trend with the amount of traps and nid, which, as discussed in the main text, can still be a consequence of energetic barriers in these devices.

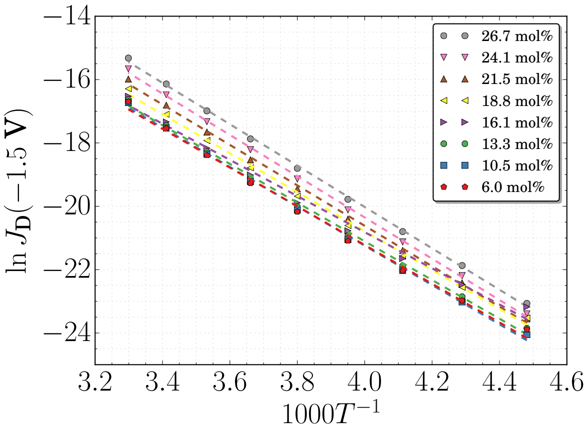


**Supplementary Figure 21: Arrhenius analysis for TPDP:C$_{60}$ at $V = -1.5$ V**

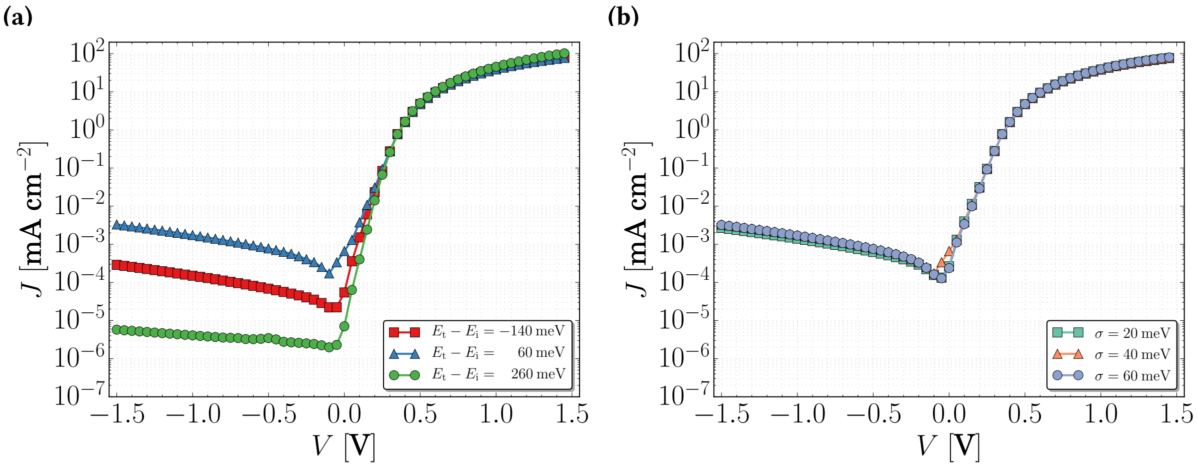


**Supplementary Figure 22: Influence of $E_t$ and $\sigma$ on the dark current of TPDP:$C_{60}$ (26.7 mol%) | (a)** Simulated for different positions of the trap level in relation to the midgap energy for this system, the latter here defined as -4.45 eV. The curve 60 meV away from midgap represents $E_t$ at -4.51 eV, as measured for this system. As $E_t$ is moved away from midgap, the contribution of traps to the dark current decreases, as predicted by by Eq. 4 of the main text. **(b)** Simulated for different widths of the trap density of states. Within the studied range, this width plays a minor role, and no strong trend can be observed when varying this parameter.

# Supplementary References


1. Barlow, S. *et al.* Synthesis, ionisation potentials and electron affinities of hexaazatrinaphthylene derivatives. *Chem.–Eur. J.* **13**, 3537–3547 (2007). DOI: 10.1002/chem.200601298.

2. Yadav, R. A. K., Dubey, D. K., Chen, S.-Z., Liang, T.-W. & Jou, J.-H. Role of Molecular Orbital Energy Levels in Oled Performance. *Sci. Rep.* **10**, 1–15 (2020). DOI: 10.1038/s41598-020-66946-2.

3. Hamwi, S. *et al.* The Role of Transition Metal Oxides in Charge-Generation Layers for Stacked Organic Light-Emitting Diodes. *Adv. Funct. Mater.* **20**, 1762–1766 (2010). DOI: 10.1002/adfm.201000301.

4. Hoppe, H. *et al.* Quality Control of Polymer Solar Modules by Lock-in Thermography. *J. Appl. Phys.* **107**, 014505 (2010). DOI: 10.1063/1.3272709.

5. McMahon, T., Berniard, T. & Albin, D. Nonlinear Shunt Paths in Thin-Film CdTe Solar Cells. *J. Appl. Phys.* **97**, 054503 (2005). DOI: 10.1063/1.1856216.

6. Dongaonkar, S. *et al.* Universality of Non-Ohmic Shunt Leakage in Thin-Film Solar Cells. *J. Appl. Phys.* **108**, 124509 (2010). DOI: 10.1063/1.351850.

7. Lüssem, B., Riede, M. & Leo, K. Doping of Organic Semiconductors. *physica status solidi (a)* **210**, 9–43 (2013). DOI: 10.1002/pssa.201228310.

8. Zheng, Y., Fischer, A., Sergeeva, N., Reineke, S. & Mannsfeld, S. C. Exploiting Lateral Current Flow Due to Doped Layers in Semiconductor Devices Having Crossbar Electrodes. *Org. Electron.* (2019). DOI: 10.1016/j.orgel.2018.10.040.

9. Walter, T., Herberholz, R., Müller, C. & Schock, H. W. Determination of Defect Distributions From Admittance Measurements and Application to Cu(In,Ga)$Se_2$ Based Heterojunctions. *J. Appl. Phys.* **80**, 4411–4420 (1996). DOI: 10.1063/1.363401.

10. Xu, L., Wang, J. & Hsu, J. W. Transport Effects on Capacitance-Frequency Analysis for Defect Characterization in Organic Photovoltaic Devices. *Phys. Rev. Appl* **6**, 064020 (2016). DOI: 10.1103/PhysRevApplied.6.064020.

11. Fischer, J. *et al.* Density of States Determination in Organic Donor-Acceptor Blend Layers Enabled by Molecular Doping. *J. Appl. Phys.* **117**, 245501 (2015). DOI: 10.1063/1.4922587.

12. Wang, S., Kaienburg, P., Klingebiel, B., Schillings, D. & Kirchartz, T. Understanding Thermal Admittance Spectroscopy in Low-Mobility Semiconductors. *The Journal of Physical Chemistry C* **122**, 9795–9803 (2018). DOI: 10.1021/acs.jpcc.8b01921.

13. Werner, F., Babbe, F., Elanzeery, H. & Siebentritt, S. Can We See Defects in Capacitance Measurements of Thin-Film Solar Cells? *Prog. Photovoltaics Res. Appl.* **27**, 1045–1058 (2019). DOI: 10.1002/pip.3196.

14. Shockley, W. & Read Jr, W. Statistics of the Recombinations of Holes and Electrons. *Phys. Rev.* **87**, 835 (1952). DOI: 10.1103/PhysRev.87.835.

15. Pieters, B. E., Decock, K., Burgelman, M., Stangl, R. & Kirchartz, T. Advanced Characterization Techniques for Thin Film Solar Cells. In Abou-Ras, D., Kirchartz, T. & Rau, U. (eds.) *Advanced Characterization Techniques for Thin Film Solar Cells*, chap. 23, 633–659 (Wiley-VCH Verlag GmbH & Co. KGaA, Weinheim, Germany, 2016), 2nd edn. ISBN: 9783527699025.

16. Welch, P. The Use of Fast Fourier Transform for the Estimation of Power Spectra: A Method Based on Time Averaging Over Short, Modified Periodograms. *IEEE Trans. Audio Electroacoust.* **15**, 70–73 (1967). DOI: 10.1109/TAU.1967.1161901.

17. Vandewal, K., Benduhn, J. & Nikolis, V. C. How to Determine Optical Gaps and Voltage Losses in Organic Photovoltaic Materials. *Sustainable Energy & Fuels* 538–544 (2017). DOI: 10.1039/C7SE00601B.

18. Mantri, P., Rizvi, S. & Mazhari, B. Estimation of Built-in Voltage from Steady-State Current–Voltage Characteristics of Organic Diodes. *Org. Electron.* **14**, 2034–2038 (2013). DOI: 10.1016/j.orgel.2013.04.030.

19. Schwarze, M. *et al.* Band Structure Engineering in Organic Semiconductors. *Science* **352**, 1446–1449 (2016). DOI: 10.1126/science.aaf0590.

20. Nell, B., Ortstein, K., Boltalina, O. V. & Vandewal, K. Influence of Dopant-Host Energy Level Offset on Thermoelectric Properties of Doped Organic Semiconductors. *The Journal of Physical Chemistry C* **122**, 11730–11735 (2018). DOI: 10.1021/acs.jpcc.8b03804.

21. Polander, L. E. *et al.* Hole-transport material variation in fully vacuum deposited perovskite solar cells. *APL Mater.* **2**, 081503 (2014). DOI: 10.1063/1.4889843.

22. Murgatroyd, P. Theory of Space-Charge-Limited Current Enhanced by Frenkel Eeffect. *J. Phys. D: Appl. Phys.* **3**, 151 (1970). DOI: 10.1088/0022-3727/3/2/308.
